# Prerequisites for Cost-Effective Home Blood Pressure Telemonitoring: Early Health Economic Analysis

**DOI:** 10.2196/64386

**Published:** 2025-05-08

**Authors:** Job van Steenkiste, Pim van Dorst, Daan Dohmen, Cornelis Boersma

**Affiliations:** 1 Department of Internal Medicine, Maasstad Hospital, Rotterdam, The Netherlands; 2 Department of Hospital Pharmacy, Erasmus MC University Medical Center, Rotterdam, The Netherlands; 3 Faculty of Management Sciences, Open University, Valkenburgerweg 177, Heerlen, 6419 AT, The Netherlands; 4 Department of Health Sciences, University Medical Center Groningen, University of Groningen, Groningen, The Netherlands; 5 Health-Ecore Ltd, Zeist, The Netherlands

**Keywords:** hypertension, blood pressure, telemonitoring, cost-effectiveness, economic evaluation, monitoring, health economics, cost, cost-effective, management, cardiovascular disease, intervention, lifestyle, adherence, clinical trials

## Abstract

**Background:**

Home blood pressure telemonitoring (HBPT) has been proposed to enhance adherence and optimize health care delivery, yet its prerequisites for cost-effective implementation remain unclear.

**Objective:**

This study aims to quantify the potential cost-effectiveness of HBPT and identify prerequisites for cost-effective implementation of HBPT in comparison to standard hypertension management, using an early health economic analysis from a societal perspective.

**Methods:**

A decision-analytic Markov model with a lifetime horizon (30 years) and a willingness-to-pay threshold of €20,000 (€1=US $1.09) per quality-adjusted life year (QALY) was developed to assess the cost-effectiveness of HBPT compared to standard of care (SOC). The HBPT intervention was based on an existing HBPT program applied by the Maasstad Hospital, Rotterdam, the Netherlands. The model incorporated 12 health states: 7 blood pressure states, 1 cardiovascular (CV) event, 1 recurrent CV event, 1 postrecurrent CV event, 1 all-cause death, and 1 CV disease–related death. A hypothetical cohort of 1000 patients (average age 65.3 years) was modeled, and results were reported in costs, QALYs, and the incremental cost-effectiveness ratio (ICER). The model assumed 3 in-person outpatient department (OPD) consultations in the SOC group and 1.5 in the HBPT group. Extensive sensitivity analyses were performed to identify important variables for the cost-effective implementation of HBPT.

**Results:**

Following the base-case analysis, HBPT was not cost-effective with an ICER of €20,386 per QALY. Sensitivity analyses indicated that reducing the number of in-person OPD consultations resulted in a more favorable ICER. Specifically, reducing the number of in-person OPD consultations to 1.48 annually resulted in an ICER below the willingness-to-pay threshold. Reducing the in-person OPD consultations to an average of 1.18 per year would make HBPT cost-saving. Scenario analyses revealed that extending the duration of HBPT’s clinical effect to 2 or 3 years substantially improved the ICER. Additionally, targeting HBPT toward patients aged 64 years or below further improved the ICER.

**Conclusions:**

HBPT could result in cost-effective or cost-saving outcomes with only minor reductions in in-person OPD consultations. These findings highlight the potential of HBPT to transform hypertension management by replacing traditional hypertension management with more efficient care using remote patient monitoring.

## Introduction

Hypertension remains one of the most important risk factors for cardiovascular diseases (CVDs) [1]. Despite lifestyle and drug therapy interventions, a significant proportion of patients with hypertension remains inadequately controlled, which is mostly the result of poor medication adherence [2]. Home blood pressure telemonitoring (HBPT) has been proposed to improve adherence [2,3] by allowing patients to measure their blood pressure at home while being remotely monitored by their health care providers. Proactive monitoring in patients with off-target blood pressures could improve overall blood pressure control through adjustment of medical treatments or by improving adherence, in particular to drug therapy [4]. Besides its potential to improve clinical outcomes, HBPT could optimize health care delivery and resource use [5] by including patient-specific measurement schedules and monitoring algorithms, designed by the responsible health care providers. Automated alerts could inform the clinician if the patient remains off-target, thereby drawing the clinician’s attention to those patients who need it the most. Furthermore, modern-day telemonitoring platforms (eg, Luscii [6] and Patient Journey App [7]) do not solely provide measuring and monitoring functionalities but also serve as a platform for digital coaching and education on lifestyle factors that can further improve clinical outcomes [3].

Recent clinical evidence on HBPT confirms positive effects on blood pressure control [8], but widespread adoption of HBPT is still limited in the Netherlands. One of the perceived barriers to large-scale implementation is the lack of a clear reimbursement structure, which is related to a lack of evidence on the cost-effectiveness of this digital health intervention [9]. Clinical trials evaluating the effectiveness of HBPT often have limited follow-up durations [8]. Consequently, they only demonstrate short-term benefits on blood pressure control and do not capture the potential long-term advantages, such as reductions in cardiovascular (CV) events. Furthermore, available evaluations of HBPT in patients with hypertension mainly focus on the cost impact of HBPT, do not report on the impact of HBPT on the quality of life of the patient [10,11], and are not representative of the Dutch hospital setting [12]. Hence, there is a need to quantify the long-term value of HBPT in terms of costs and health outcomes while considering the limited data availability on resource use and effectiveness.

In this study, we aim to quantify the potential of HBPT in terms of cost-effectiveness with an early health economic analysis in patients with hypertension. Additionally, we aim to identify important prerequisites for cost-effective implementation of HBPT.

## Methods

### Study Design

**Figure 1.**
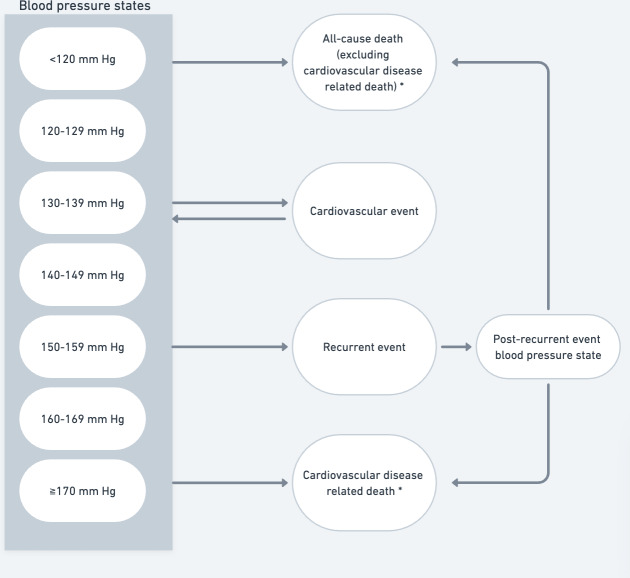
Markov model including 12 different health states. *Risk of all-cause death and cardiovascular disease–related death were blood pressure independent.

This early health economic evaluation is reported per the 2022 CHEERS (Consolidated Health Economic Evaluation Reporting Standards) guidelines for reporting economic evaluations (Checklist 1). Given the lack of long-term efficacy data of HBPT [8] and the resulting uncertainty in the clinical evidence, the current evaluation is considered an early health economic evaluation, which is based on available literature [13]. A decision-analytic Markov model (see Figure 1) with a lifetime (30 years) horizon and a willingness-to-pay (WTP) threshold of €20,000 (€1=USD $1.09) per quality-adjusted life year (QALY) [14], to assess the cost-effectiveness of HBPT in combination with drug therapy for patients with hypertension. A societal perspective was applied (eg, including direct medical costs and nonmedical costs) according to the Dutch guideline for conducting health economic research [15]. All costs were inflated using Dutch inflation rates to reflect the costs in 2024 euro [16]. The model was developed in R statistical software version 4.4.1 [17].

### Model Overview

#### Structure

The model included a hypothetical population of 1000 patients with an average age of 65.3 years and consisted of 12 health states: <120, 120‐129, 130‐139, 140‐149, 150‐159, 160‐169, ≥170 mm Hg, CV event, recurrent event, postrecurrent event, all-cause death, and CVD-related death. The initial distribution and demographics (Multimedia Appendix 1 [8,18-23]) of patients over the systolic blood pressure states were based on the Blood Pressure Lowering Treatment Trialists’ Collaboration study [18], reflecting a real-world distribution of patients with hypertension and no history of CVD. Patients could transition from higher blood-pressure states to lower blood-pressure states on an annual basis, based on drug therapy, until the patients were on target (120‐129 mm Hg). A reverse transition was not possible. Each year, patients could experience a CV event, after which the patients returned to the pre-event blood-pressure health state, as no direct blood pressure lowering effect due to the CV event was expected. A CV event was a composite event consisting of either a myocardial infarction (MI), a cerebral hemorrhage, or an ischemic cerebrovascular event, and the event risk was blood pressure dependent [19]. Patients could experience a recurrent event after which they progressed into the postrecurrent event health state. The risks of all-cause death and CVD-related death were assumed to be blood pressure independent.

#### Standard of Care

Standard of care (SOC) was based on the current practice of hypertension management in the Netherlands. The care provided via the hospital outpatient department (OPD) was based on the latest European Society of Hypertension guidelines on the management of arterial hypertension [24]. In the model, patients in the SOC group would be managed with drug therapy and lifestyle interventions. Patients would on average have 3 in-person OPD consultations in the hospital with their clinician during each 1-year cycle, based on the standard diagnosis-treatment combination for patients with hypertension in the Netherlands [25].

#### Intervention

Patients in the HBPT group were similarly managed in terms of drug treatment compared to the SOC group. The HBPT intervention was based on the HBPT program developed by the Maasstad Hospital in Rotterdam, the Netherlands, and adopted and studied throughout the region [26]. In this program, which is conducted in a hospital setting, patients measured their blood pressure during a complete week with 2 measurements in the morning and 2 in the evening. Measurement weeks were scheduled depending on the level of blood pressure control (eg, weekly in case of very uncontrolled blood pressure [>180/110 mm Hg] and monthly in case of controlled blood pressure [<140/90 mm Hg]), but on average occurred once every month. The monitoring platform used was the Luscii [6] application, which is the most widely used platform in the Netherlands for remote patient monitoring. The most frequently used patient monitoring setup in the Netherlands includes a “hospital-based telemonitoring center” with specialized e-nurses. Blood pressure data are automatically synchronized via the monitoring platform to a special health care provider dashboard, integrated into the electronic health record. The e-nurses in the telemonitoring center assess all the alarms generated by the monitoring platform based on the blood pressure data and discuss these alarms with clinicians if needed. A schematic overview of the HBPT processing steps is included in Multimedia Appendix 2. The clinicians supervising the e-nurses are internal medicine specialists, residents, or nurse practitioners who would also be involved in the SOC for patients with hypertension. They are also responsible for remotely adjusting blood pressure medication if needed.

### Model Input Parameters

#### Probabilities and Efficacy Input

Multimedia Appendix 1 provides an overview of the baseline blood pressure distribution, annual event probabilities, and efficacy model inputs. Each systolic blood pressure state corresponded to a risk of a CV event, which was based on a large prospective real-world study [19]. The transition probability from a higher blood pressure state to a lower blood pressure state was based on a decrease of 5.1 mm Hg per year, which corresponded to the clinical effect of pharmacological therapy reported in the latest available meta-analysis [20] and applied to both groups. Patients in the HBPT group had an additional decrease of 12 mm Hg in systolic blood pressure in the first year due to HBPT. This additional effect was based on the latest available literature on the clinical effectiveness of HBPT [8]. A notable proportion, 19.7% of the patients had resistant hypertension resulting in the absence of blood pressure reduction [21]. The probability of dying from a CV event (CVD-related death) was based on the Blood Pressure Lowering Treatment Trialists’ Collaboration study [18]. The probability of all-cause mortality was based on the age-based population mortality in the Netherlands [22] and was corrected for CVD-related deaths [22]. The probability of suffering a recurrent CV event was derived from a large study assessing the 10-year risk of recurrent vascular events [23].

#### Utilities

The utilities of the model health states were derived from published literature (Multimedia Appendix 3 [27-30]). The baseline utility value was 0.96 for patients with hypertension [27], which declined to 0.79 following an MI [28], 0.64 following a cerebral infarction [28], and 0.59 following an intracranial hemorrhage [29]. The weighted average of these event utilities was 0.67 and was used in the model as the “postevent” utility for the year after the event occurred. The conservative assumption was made that after 1 year of the event, the utility would equal the baseline utility.

A recurrent intracerebral infarction or MI corresponded with a utility of 0.74 and 0.62, respectively [30]. For a recurrent intracranial hemorrhage, the utility value was considered equal to the utility value of a first intracranial hemorrhage, which was 0.59 [29]. The weighted average utility of a recurrence was 0.64 and was used for the year the recurrent event occurred and the subsequent years the patient was in the postrecurrent health state [27].

#### Costs and Discounting Rates

Costs were divided into direct medical costs and nonmedical costs (Multimedia Appendix 4 [16,25,31-42]). For the HBPT group, direct medical costs consisted of a one-time out-of-pocket purchase of a blood pressure device [31], costs for remote monitoring [32], standard drug costs [33,43], additional drug costs [44], and in-person OPD consultations. The remote monitoring costs were based on an official Dutch tariff [32] for patients who are part of a remote monitoring program. A hospital can claim this tariff 3 times a year as a flat fee for a patient who is remotely monitored to cover costs for the license of telemonitoring software, salaries for the involved health care workers, and development costs. In the SOC group, direct medical costs only consisted of standard drug costs and costs for the in-person OPD consultations. Direct medical costs for a stroke (infarction and hemorrhage), MI, or CV-related death were based on data available from the Dutch National Health Care Institute and Ministry of Health, Welfare and Sport [34,35]. Total costs for each event were based on the overall reported expenses divided by the weighted incidences of both stroke and MI.

Nonmedical costs consisted of travel costs, parking costs, and costs related to productivity losses in both the SOC and HBPT groups. Productivity losses were based on work absence resulting from the in-person OPD consultations (1 hour for each visit) or due to an event (17.7 absent working days) and were based on data from the Dutch National Healthcare Institute [36] and the Trimbos Institute [37]. The costs of productivity losses were based on the average labor participation [38], average hourly wage [39], and average working week in the 65‐75 years age group corresponding with the average age of 65.3 years used in the current analysis (based on the Blood Pressure Lowering Treatment Trialists’ Collaboration study [18]. Friction costs following a death were calculated based on the friction costs method [40].

Discounting rates were 3% for the costs and 4% for the health outcomes based on the Dutch Economic Evaluation guidelines [15].

### Outcomes

The outcome measures used to compare the 2 interventions in this study were costs, QALYs, and incremental cost-effectiveness ratios (ICERs) presented as cost per QALY gained.

### Univariate Sensitivity Analysis and Scenario Analysis

To assess the impact of uncertainty on the ICER, an extensive sensitivity analysis was performed for the current early health economic analysis.

A univariate sensitivity analysis was performed to quantify the impact of parameter uncertainty on the ICER by varying all individual parameters one by one with ±20% of the mean. For utilities, the upper limit was restricted to a maximum of 1. In addition to the univariate sensitivity analysis, three scenario analyses were performed. (1) Since telemonitoring was expected to result in a reduction in the number of OPD consultations, the interdependency between these variables was assessed. The ICER was calculated for a range of telemonitoring costs and a range of frequencies of OPD consultations. (2) A scenario with a prolonged clinical effect of HBPT (2 and 3 years compared to 1 year in the base case) was modeled to assess the potential effect on the ICER. (3) To assess the impact of age on the ICER, the age at which remote patient monitoring is started was modeled over a range of 30 to 75 years.

### Assumptions

The following assumptions were made during model development. (1) A proportion of 19.7% of patients with hypertension was considered to have resistant hypertension [21]. About half of these patients have so-called “apparent resistant hypertension,” which is antihypertensive treatment failure due to drug nonadherence. We assumed that the HBPT intervention prevents nonadherence, resulting in only 9.85% of the patients having resistant hypertension in the HBPT group. (2) Patients receiving HBPT will have 50% fewer in-person consultations with their clinician or specialist nurse, as the remote patient monitoring partially replaces the need for in-hospital blood pressure measurements and identifies on-target patients who might not require a regular follow-up consultation. It was assumed that the patients in the HBPT group would on average have 1.5 in-person OPD consultations annually. (3) Since most of the HBPT trials have follow-up durations of up to 1 year, we assumed that HBPT would only cause an additional blood pressure lowering effect (in addition to the effect of drug therapy) in the first year (cycle 1). (4) HBPT prevents patients from suffering from overtreatment (blood pressure <120 mm Hg), which also results in an increased risk for CV events and death. Therefore, in the SOC group, patients could transition to the <120 mm Hg health state for a maximum of 1 year after which they returned to the 120‐129 mm Hg health state. In the HBPT group, it was assumed that patients could not transition to the <120 mm Hg health state. (5) The second year after a CV event, patients will return back to the baseline utility.

### Ethical Considerations

No ethics approval was applied for this study as this study was not conducted on newly generated real-world data from human participants. Data for the probabilities, costs, and utilities were derived from the available literature or from publicly available government sources.

## Results

### Base Case

In the base case, the cost for the HBPT group was €20,463,881 and €19,196,847 in the SOC group, resulting in incremental costs for HBPT of €1,267,034 compared to SOC. Additionally, HBPT resulted in 13,401.19 QALYs compared to 13,339.04 QALYs in the SOC group, resulting in an incremental effect of 62.15 QALYs in favor of HBPT. The resulting ICER for the base-case analysis was €20,386 per QALY. Based on the WTP threshold of €20,000 per QALY [14], telemonitoring is not considered cost-effective following the assumptions of the base-case analysis. The additional costs of telemonitoring outweigh the QALYs gained because of prevented first and recurrent CV events.

### Univariate Sensitivity Analysis

**Figure 2.**
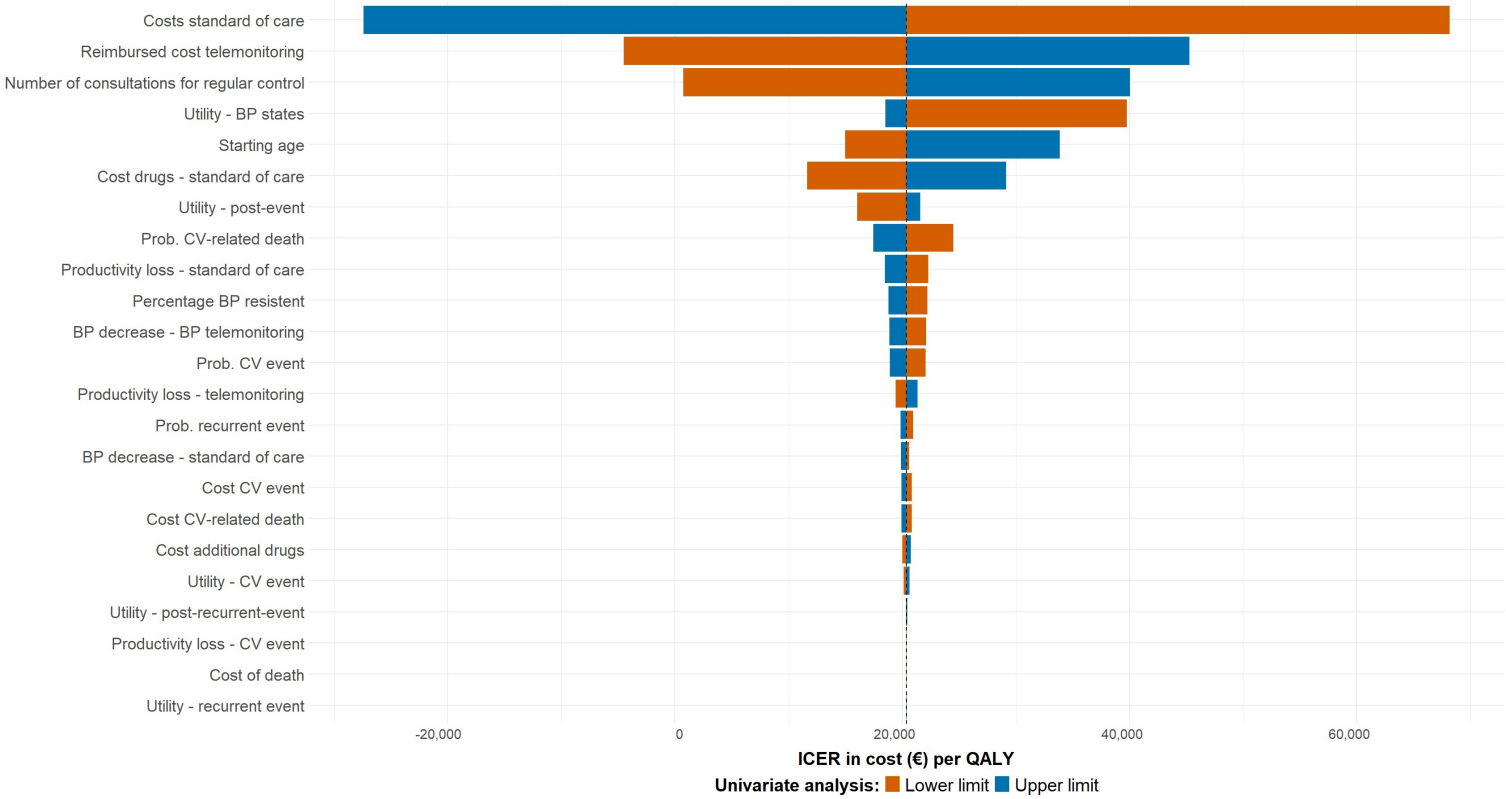
Tornado diagram of the results of the univariate sensitivity analysis (€1=US $1.09). BP: blood pressure; CV: cardiovascular; ICER: incremental cost-effectiveness ratio; Prob: probability; QALY: quality-adjusted life year.

The results of the univariate sensitivity analysis indicate the impact of parameter uncertainty on the ICER. Based on the results in Figure 2, the uncertainty in the additional costs of SOC resulting from in-person OPD consultations has the highest impact on the ICER. In case the upper limit was selected for the SOC costs (€1166.33), HBPT became cost-saving compared to SOC. With the lower limit of the reimbursed costs for telemonitoring (€403.20 instead of €504.00 in the base case), HBPT also became cost-saving compared to SOC. In case the number of consultations was reduced to 1.2 per year in the HBPT group, the ICER dropped to €742 per QALY and HBPT was considered cost-effective. In all other cases, the analysis of parameter uncertainty resulted in an ICER between €11,641 per QALY and €39,758 per QALY.

### Scenario Analysis

#### Scenario 1: Variable Telemonitoring Costs and Frequency of OPD Consultations

**Figure 3.**
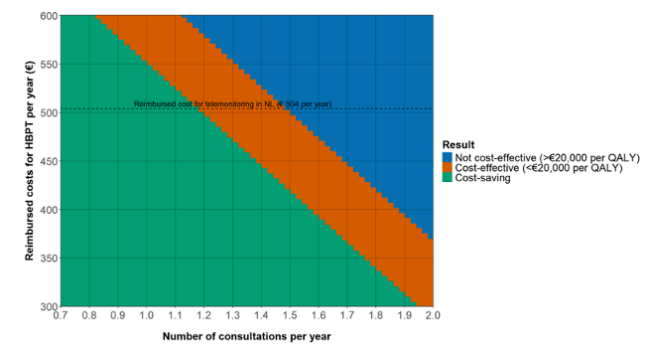
ICER results (not cost-effective, cost-effective, and cost-saving) of scenario analysis calculated over a range of costs for HBPT per year and a range of in-person consultations per year (€1=US $1.09). HBPT: home blood pressure telemonitoring; ICER: incremental cost-effectiveness ratio; QALY: quality-adjusted life year.

One important assumption of the current model is related to the number of in-person consultations for patients in the HBPT group. It was assumed that in the HBPT group, the number of annual consultations dropped from 3 to 1.5 per year. A further decrease in the number of consultations could result in a further reduction of the ICER. Based on the results of scenario 1, HBPT will become cost-effective (<€20,000 per QALY) with the current reimbursement of €504 per year at 1.48 in-person OPD consultations per year and will become cost-saving at 1.18 in-person OPD consultations per year (Figure 3).

#### Scenario 2: Prolonged Clinical Effect of HBPT

The duration of the effect of HBPT comes with great uncertainty and was assumed to last for only 1 year in the base-case analysis, which could be considered conservative. Scenario 2 indicates that the ICER will reach €11,154 per QALY in case the effect of HBPT lasts for 2 years (a total blood pressure reduction of 24 mm Hg after 2 years) and further declines to €9204 per QALY in case HBPT reduces the blood pressure with 12 mm Hg for 3 years (a total blood pressure reduction of 36 mm Hg after 3 years).

#### Scenario 3: Variable Starting Age HBPT

**Figure 4.**
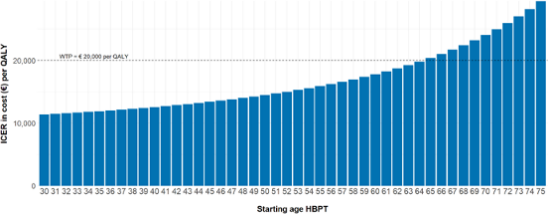
ICER per age at which HBPT is started (€1=US $1.09). HBPT: home blood pressure telemonitoring; ICER: incremental cost-effectiveness ratio; QALY: quality-adjusted life year; WTP: willingness to pay.

The results of scenario 3, in which the model was run over an age range of 30 to 75 years (Figure 4), indicate that the younger the patient’s age at the start of HBPT, the lower the ICER. If HBPT is started at the age of 64 years or below, HBPT could be considered a cost-effective intervention.

## Discussion

### Principal Findings

#### Overview

To the best of our knowledge, this is the first study to provide an early cost-effectiveness analysis of HBPT in patients with hypertension without previous CV events. Based on the current early cost-effectiveness model, which reflects a societal perspective and includes both short- and long-term costs and benefits, HBPT showed the potential to be cost-effective following realistic reductions in SOC for patients with hypertension. Specifically, a reduction in the number of OPD consultations in the HBPT group will make HBPT cost-effective or even cost-saving. These findings underscore the potential of HBPT and with that the importance of genuine digital transformation in health care, advocating for the substitution of traditional OPD care with digital and remote care, rather than providing digital care as an add-on to standard OPD care. Additionally, we found that early (ie, younger age) and sustained telemonitoring further improves the cost-effectiveness of HBPT.

#### Outcome-Based Health Care

Policy makers should make use of the fact that HBPT can be cost-effective following a reduction in the number of OPD consultations. The current reimbursement structure provided by the Dutch Health Care Authority [32] does not include any requirements in terms of reducing standard care and therefore seems unsuitable in its current form. Outcome-based health care contracts [45], characterized by performance fees linked to predefined shared objectives between hospitals and insurance companies, are ideally suited for HBPT to pursue genuine digital transformation efforts. By setting a shared objective in terms of physical care replacement with HBPT, a sustainable system could be established that allows for efficient (with fewer resources) and cost-effective care delivery along the lines of value chain optimization and significant displacement effects.

#### The Current Business Model for HBPT

Hypothetically, HBPT should be able to realize short-term benefits through greater efficiency with regard to care organization and long-term benefits, resulting from a greater level of blood pressure control, which translates into a reduction in CV events and CV-related deaths. We found that short-term benefits realized through a substantial reduction in OPD consultations had a major impact on the ICER, but the impact of long-term benefits appeared to be limited. The 1-year effect of HBPT on the blood pressure of patients resulted in minor between-group differences in CV events or CVD-related deaths. Extending the effect of HBPT to 2 or 3 years substantially reduced the ICER, but clinical evidence supporting this assumption is lacking, as follow-up durations in clinical trials are usually no longer than 12 months [8]. Therefore, short-term benefits resulting from more efficient care delivery are expected to become the major driver for a sustainable business model for HBPT. The further upward potential of remote monitoring comes with a multimorbidity perspective (eg, hypertension and diabetes). Many patients have a variety of comorbidities and multiple consultations with different specialists. If one remote monitoring program reduces the number of consultations across multiple medical specialties, remote monitoring is more likely to result in cost savings.

#### Alternative Models Available in the Literature

The only available comparable study [46] evaluates the cost-effectiveness of HBPT in a poststroke population using a Markov cohort simulation. The HBPT intervention was cost-saving in the base case and cost-effective in the scenario analyses with an ICER of US $1200-4700 per QALY. In contrast to our study, the benefit in terms of blood pressure reduction due to HBPT was modeled as a continuous effect (year after year). Even though our scenario analyses with 2 and 3 years of clinical benefits of HBPT resulted in HBPT being cost-effective, the results of the previously described study [46] should be considered as optimistic as evidence on a sustained (year after year) effect is lacking. Other studies that reported a positive effect of remote monitoring include heart failure monitoring [47-49] or monitoring of patients with COVID-19 [50]. These studies [46,47,50] highlight the importance of reducing short-term care consumption to come to a favorable ICER. This advocates for the substitution of traditional care with digital and remote care, rather than providing digital care as an add-on to standard care.

### Limitations

The current early health economic analysis comes with limitations that should be considered when interpreting the results of this study. First, we did not consider any effects of HBPT on diastolic blood pressure, which could have resulted in potential CVD risk reduction. However, since most of the hypertensive population has systolic or both systolic and diastolic hypertension, the impact of this simplification is expected to be limited.

Second, baseline blood pressure distribution and CVD-related death were derived from one study, which could impact the generalizability of the results [19]. However, given the large number of patients included in the study (n=96,268) and the follow-up period of 10 years, the study was considered highly valuable for the current early health economic analysis. Gathering country-specific data on the baseline blood pressure distribution and CVD-related death will become important when the current model is applied to inform reimbursement decisions.

Third, as many patients with hypertension often have other relevant comorbidities, reducing the number of in-person visits during the HBPT program might negatively impact the provided care and cost-effectiveness for other relevant diseases, which would normally be addressed during the same consultation. It appears to be more likely, however, that future remote patient monitoring programs will encompass multiple conditions (eg, hypertension and diabetes) and thereby overcome this potential disadvantage.

Fourth, the model does not allow patients to move to higher systolic blood pressure states, which could result in an overestimation of long-term blood pressure regulation. This limitation was partially overcome by classifying part of the population as apparently resistant, implying their blood pressure did not decrease. Furthermore, any potential overestimation would affect both the SOC and HBPT groups, thus largely neutralizing the impact on comparative results.

Future research should focus on reducing uncertainty on key input parameters, which include the duration of the effect and the number of OPD consultations per year needed in addition to HBPT. Additionally, future research should focus on the effect of scale in terms of the number of patients included in the HBPT program as an additional prerequisite for sustainable implementation, as the one-time investment costs are substantial when starting with HBPT. Moreover, this model should be validated with real-world data, specifically from a Dutch randomized trial. Finally, future research should consider the cost-effectiveness across different care settings, as a significant portion of patients with hypertension are treated by general practitioners.

### Conclusion

Based on the current early health economic analysis, we found HBPT to be cost-effective, provided it will result in a genuine digital transformation in health care and thereby substantially reduce the number of standard OPD consultations.

## Supplementary material

10.2196/64386 Multimedia Appendix 1 Baseline blood pressure distribution, annual event probabilities, and efficacy model inputs.

10.2196/64386 Multimedia Appendix 2 Telemonitoring organization and alert processing.

10.2196/64386 Multimedia Appendix 3 Health utility values.

10.2196/64386 Multimedia Appendix 4 Costs and discounting rates.

10.2196/64386 Checklist 1 CHEERS (Consolidated Health Economic Evaluation Reporting Standards) 2022 checklist.

## Abbreviations

CHEERS: Consolidated Health Economic Evaluation Reporting Standards
CV: cardiovascular
CVD: cardiovascular disease
HBPT: home blood pressure telemonitoring
ICER: incremental cost-effectiveness ratio
MI: myocardial infarction
OPD: outpatient department
QALY: quality-adjusted life year
SOC: standard of care
WTP: willingness to pay
