# Supplementary material for: Prerequisites for Cost-Effective Home Blood Pressure Telemonitoring: Early Health Economic Analysis
Source: JMIR Cardio. 2025 May 8;9:e64386. doi: 10.2196/64386 (PMC12080967; doi:10.2196/64386)
Supplement: Multimedia Appendix 1 [file cardio-v9-e64386-s001.docx]

**Baseline blood pressure distribution, annual event probabilities and efficacy model inputs**

Table 1. Baseline blood pressure distribution, annual event probabilities and efficacy model inputs

| **Parameter** | **Value** | **Source** |
| --- | --- | --- |
| Baseline distribution of systolic blood pressure values (mmHg) |  |  |
| <120 | 2.6% | [18] |
| 120-129 | 5.4% | [18] |
| 130-139 | 10.1% | [18] |
| 140-149 | 16.8% | [18] |
| 150-159 | 17.0% | [18] |
| 160-169 | 20.8% | [18] |
| ≧170 | 27.3% | [18] |
| Annual probabilities for suffering a CV event for each systolic blood pressure category (mmHg) |  |  |
| <120 | 0.00888712 | [19] |
| 120-129 | 0.00631655 | [19] |
| 130-139 | 0.00684717 | [19] |
| 140-149 | 0.00973564 | [19] |
| 150-159 | 0.00973564 | [19] |
| 160-169 | 0.00973564 | [19] |
| ≧170 | 0.00973564 | [19] |
| Annual probabilities for CV disease related death |  |  |
| <120 | 0.0035935 | [18] |
| 120-129 | 0.0029955 | [18] |
| 130-139 | 0.0029955 | [18] |
| 140-149 | 0.0033942 | [18] |
| 150-159 | 0.0033942 | [18] |
| 160-169 | 0.0051865 | [18] |
| ≧170 | 0.0051865 | [18] |
| Annual probabilities for all-cause death (excluding CV disease related death) |  |  |
| Age-based annual mortality | CBS | [25] |
| Annual probabilities for recurrent event |  |  |
| Recurrent stroke (infarction/hemorrhage) | 0.01158574 | [26] |
| Recurrent MI | 0.02539163 | [26] |
| Recurrent event (composite) | 0.01846044 | [26] |
| HR post-event CV disease related death | 3 | [26] |
| Efficacy model inputs |  |  |
| Annual reduction in systolic blood pressure due to drug therapy | -5.1 mmHg | [23] |
| Annual reduction in systolic blood pressure due to HBPT, 1 year^A^ | -12 mmHg | [8] |
| Annual % of patients with resistant hypertension | 19.7% | [24] |

Abbreviations: CV = cardiovascular, MI = myocardial infarction. ^A^Since most of the HBPT trials have follow-up durations up to 1 year, we assumed that HBPT would only cause an additional blood pressure lowering effect (in addition to the effect of drug therapy) in the first year (cycle 1).
