# Supplementary material for: Prerequisites for Cost-Effective Home Blood Pressure Telemonitoring: Early Health Economic Analysis
Source: JMIR Cardio. 2025 May 8;9:e64386. doi: 10.2196/64386 (PMC12080967; doi:10.2196/64386)

**Telemonitoring organization and alert processing**

Patients measure their blood pressure at home using a validated Bluetooth-connected blood pressure machine. These measurements can trigger simple, complex or inactive/overdue alerts. A simple alert is triggered during a one-off very high or very low blood pressure or heart rate. A complex alert is triggered is a series of blood pressure or heart rate measurements are off target. Inactive and overdue alerts are triggered if the patient fails to perform the required measurement (overdue alert) or if the patients is inactive for a longer period (inactive alert). These alerts are either processed automatically by the telemonitoring platform or manually by the E-nurse in the hospital. Alerts are processed on a daily basis for each patient and could result in an administrative- or clinical action. Feedback to the patients is provided automatically by the telemonitoring platform (e.g. a protocol switch) or manually by the E-nurse (e.g. a treatment adjustment). Clinical actions are performed by Internal Medicine Specialists, residents or nurse-practitioners.


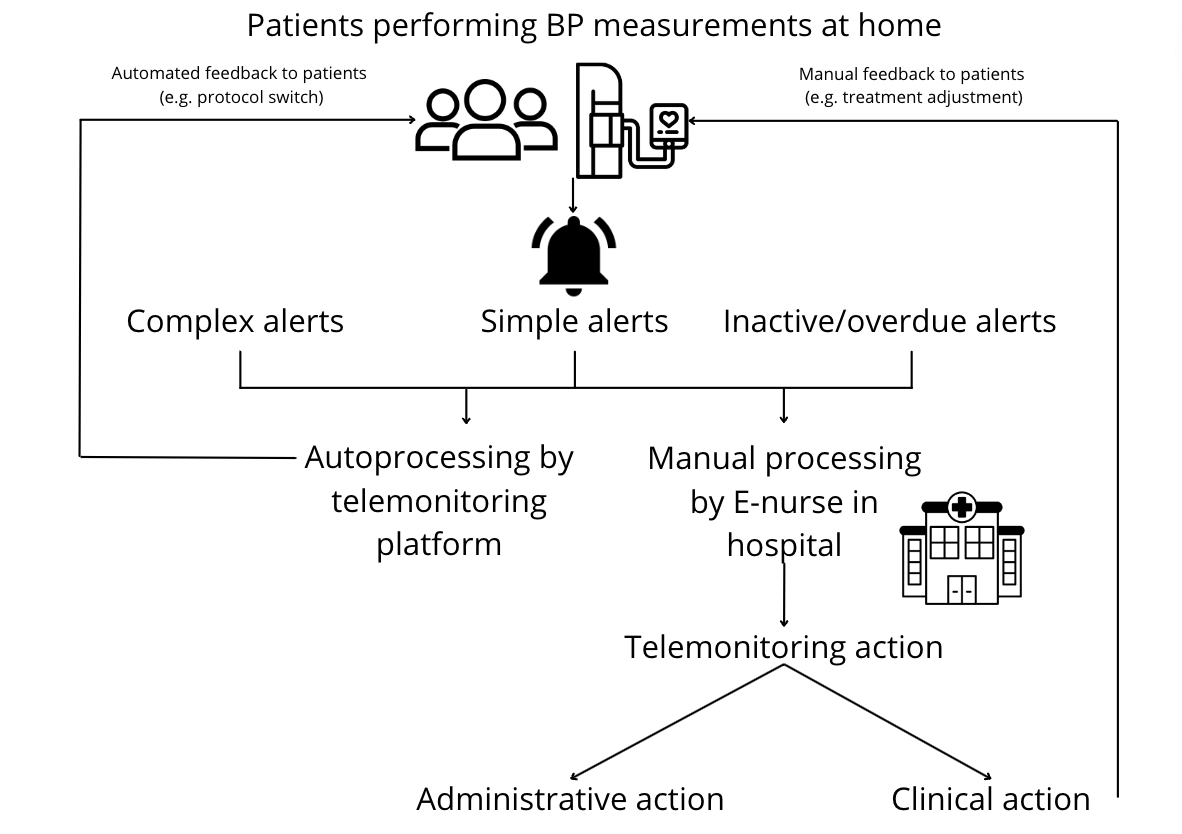

Supplement: Multimedia Appendix 2 [file cardio-v9-e64386-s002.docx]
