# Supplementary material for: Prerequisites for Cost-Effective Home Blood Pressure Telemonitoring: Early Health Economic Analysis
Source: JMIR Cardio. 2025 May 8;9:e64386. doi: 10.2196/64386 (PMC12080967; doi:10.2196/64386)
Supplement: Multimedia Appendix 3 [file cardio-v9-e64386-s003.docx]

**Health utility values**

| **Parameter** | **Value** | **Source** |
| --- | --- | --- |
| Health utility values (QoL) |  |  |
| Hypertension (baseline) | 0.96 | [27] |
| Post MI | 0.79 | [28] |
| Post cerebral infarction | 0.64 | [28] |
| Post intra-cranial hemorraghe | 0.59 | [29] |
| Post-event (composite) | 0.67 | — |
| Recurrent MI | 0.74 | [30] |
| Recurrent cerebral infarction | 0.62 | [30] |
| Recurrent intra-cranial hemorraghe | 0.59 | [29] |
| Recurrent event (composite) | 0.64 | — |

Abbreviations: MI = myocardial infarction, QoL = Quality of Life
