# Supplementary material for: Prerequisites for Cost-Effective Home Blood Pressure Telemonitoring: Early Health Economic Analysis
Source: JMIR Cardio. 2025 May 8;9:e64386. doi: 10.2196/64386 (PMC12080967; doi:10.2196/64386)
Supplement: Multimedia Appendix 4 [file cardio-v9-e64386-s004.docx]

**Costs and discounting rates**

| **Parameter** | **Value** | **Source** |
| --- | --- | --- |
| Direct medical costs |  |  |
| *HBPT* |  |  |
| BP Device | € 44.95 | [31] |
| HBPT costs (3x) | € 504.00 | [32] |
| Standard drug costs^A^ | € 176.94 | [34] |
| Additional drug costs^B^ | € 7.30 | [43] |
| Physical visits (DTC, 1.5x) | € 397.50 | [21] |
| **Total per patient per year** | **€ 1130.69** |  |
|  |  |  |
| *SOC* |  |  |
| Usual care (DTC, 3x) | € 795.00 | [21] |
| Standard drug costs^A^ | € 176.94 | [34] |
| **Total per patient per year** | **€ 971.94** | - |
|  |  |  |
| *Both groups* |  |  |
| CV event^C^ | **€ 21,821.02** | [36, 37] |
| CV disease-related death (fatal event) | **€ 21,821.02** | [36, 37] |
| Direct non-medical costs |  |  |
| *HBPT* |  |  |
| Travel + parking (1.5x) | € 11.85 | [38] |
| Productivity loss (work)^D^ | € 7.70 | [40, 41, 44] |
| **Total per patient per year** | **€ 19.55** | - |
|  |  |  |
| *SOC* |  |  |
| Travel + parking (3x) | € 23.70 | [38] |
| Productivity loss (work) | € 15.41 | [40, 41, 44] |
| **Total per patient per year** | **€ 39.11** | - |
|  |  |  |
| *Stroke* |  |  |
| Work absence^E^ | **€ 727.35** | [39] |
|  |  |  |
| *Ischemic heart disease* |  |  |
| Work absence^E^ | **€ 727.35** | [39] |
|  |  |  |
| *Death* |  |  |
| Friction costs | **€ 2010.62** | [42] |
| Discounting |  |  |
| Annual discount rate costs | 4% | [16] |
| Annual discount rate benefits | 3% | [16] |

Abbreviations: BP = blood pressure, CV = cardiovascular, DTC = Diagnosis Treatment Combination, HBPT = home blood pressure telemonitoring, SOC = standard of care.
^A^Total annual prescriptions for antihypertensives / number of patients * average prescription costs; ^B^ 0.5 additional daily tablets * costs for standard daily dose of amlodipine treatment * 365 days; ^C^weighted average of cerebral infarction, cerebral hemorrhage, and myocardial infarction; ^D^1 hour * number of appointments * % labor participation; ^E^ 17.7 absent days * average working hours per day * hourly wage.
